# Exploring Exhaled Breath Analysis in Adults With Chronic Visceral Acid Sphingomyelinase Deficiency to Identify Potential Biomarkers of Pulmonary Involvement

**DOI:** 10.1002/jimd.70039

**Published:** 2025-07-27

**Authors:** Eline C. B. Eskes, Bauke V. Schomakers, Michel van Weeghel, Suzanne W. J. Terheggen‐Lagro, Lilian J. Meijboom, Carla E. M. Hollak, Paul Brinkman, Barbara Sjouke

**Affiliations:** ^1^ Department of Endocrinology and Metabolism Amsterdam UMC Location University of Amsterdam Amsterdam the Netherlands; ^2^ Inborn Errors of Metabolism Amsterdam Gastroenterology Endocrinology Metabolism Amsterdam the Netherlands; ^3^ Laboratory Genetic Metabolic Diseases Amsterdam UMC Location University of Amsterdam Amsterdam the Netherlands; ^4^ Core Facility Metabolomics Amsterdam UMC Location University of Amsterdam Amsterdam the Netherlands; ^5^ Emma Children's Hospital Department of Pediatric Pulmonology and Allergy Amsterdam UMC Location University of Amsterdam Amsterdam the Netherlands; ^6^ Department of Radiology and Nuclear Medicine Amsterdam UMC, Location Vrije Universiteit Amsterdam Amsterdam the Netherlands; ^7^ Amsterdam Cardiovascular Sciences Pulmonary Hypertension and Thrombosis Amsterdam the Netherlands; ^8^ Department of Pulmonary Medicine Amsterdam UMC Location University of Amsterdam Amsterdam the Netherlands; ^9^ Amsterdam Institute for Infection and Immunity Amsterdam the Netherlands; ^10^ Department of Internal Medicine Radboud UMC Nijmegen the Netherlands

**Keywords:** acid sphingomyelinase deficiency, biomarkers, exhaled breath

## Abstract

Acid sphingomyelinase deficiency (ASMD) is a rare lysosomal storage disease. The most commonly affected organs are the spleen, the liver, and the lungs. Pulmonary involvement resembles interstitial lung disease and often leads to decreased diffusion capacity of the lungs for carbon monoxide (DLCO). An emerging technique in pulmonary research is the analysis of exhaled breath. The aim of this study was to investigate potential markers of pulmonary involvement in the exhaled breath of adult chronic visceral ASMD patients and to quantify findings on high‐resolution computed tomography (HRCT) of the lungs in order to be able to correlate HRCT findings with (the potential) markers for pulmonary involvement. Fifteen adult, chronic visceral ASMD patients and 34 age‐, sex‐, and smoking habit‐matched healthy controls were recruited and provided two different types of exhaled breath samples: exhaled air and exhaled condensate. Additionally, pulmonary function testing was performed for both patients and healthy controls, and HRCT of the lungs and biochemical markers were available for patients. Exhaled breath samples were analyzed using gas and liquid chromatography‐mass spectrometry (GC–MS and LC–MS respectively). Fifteen compounds of interest were identified based on significant differences between ASMD patients and healthy controls, of which the most promising were 2‐hydroperoxyhexane, 6‐heptyn‐2‐one, and 4‐pentenyl acetate. Other compounds have been described in the context of systemic sclerosis (i.e., acetophenone) or lung cancer (i.e., benzaldehyde and dodecane). Some markers were associated with the pathophysiological process of lipid peroxidation (i.e., decane, dodecane and 2‐methylnonane). SPLSDA and AUROCC analyses showed that the model was better able to distinguish the patients with pulmonary involvement from their matched controls than all patients from all controls. Lastly, a quantitative HRCT score was performed and correlated with patients' DLCO (R = −0.74, *p* = 0.006). The most promising markers based on our analyses (i.e., 2‐hydroperoxyhexane, 6‐heptyn‐2‐one and 4‐pentenyl acetate) have not been described in previous studies in the pulmonary field and might be ASMD‐specific.

## 1 Introduction

Acid Sphingomyelinase Deficiency (ASMD, OMIM 607616), also known as Niemann‐Pick disease types A and B, is a rare lysosomal storage disorder caused by two bi‐allelic mutations in the *SMPD1* gene resulting in a deficiency of acid sphingomyelinase (EC 3.1.4.12) [1, 2]. This enzyme converts sphingomyelin into ceramide; hence, a deficiency leads to sphingomyelin accumulation, which is mainly observed in macrophages. The disease covers a broad clinical spectrum, from severely affected infants with visceral and neurological manifestations lethal in early childhood (the acute neurovisceral subtype) to mildly affected adults without neurological manifestations (the chronic visceral subtype) [3, 4]. The most commonly affected organs are the spleen, liver, and lungs, with hepatosplenomegaly and decreased diffusion capacity being the most common visceral manifestations [5, 6].

Pulmonary involvement of ASMD mostly resembles interstitial lung disease (ILD) [7]. Accumulation of sphingomyelin and foamy macrophages is observed in the alveolar space, inter‐ and intralobular septae, and pleura [8]. On pulmonary imaging, thickened inter‐ and intralobular septae, as well as ground‐glass opacities, are often observed [9]. Most patients have decreased diffusion capacity of the lungs for carbon monoxide (DLCO) [5, 6].

Pulmonary manifestations in ASMD vary greatly, with on the one hand patients showing normal or mildly decreased DLCO without functional impairment and on the other hand patients with severely decreased DLCO causing complaints of dyspnea and exercise intolerance with limitations in daily functioning, possibly with (partial) oxygen dependency [5, 10, 11, 12]. The limitation of daily activities due to pulmonary manifestations in a subgroup of ASMD patients is one of the most debilitating features of the disease [6, 13]. However, predisposing factors for severe pulmonary involvement of ASMD were not identified in previous natural history studies [5, 6, 12].

Treatment with enzyme replacement therapy (ERT) showed a positive effect on pulmonary involvement in an international clinical phase II/III trial. Treatment during one year resulted in an increase of 22%–28% of predicted DLCO and amelioration of pulmonary imaging with significant improvement of ground glass opacities [14, 15]. ERT for the visceral manifestations of ASMD (olipudase alfa TM, Sanofi Genzyme) has been approved by the European and US authorities recently [16, 17]. To aid in selecting patients who will benefit from therapy, several aspects regarding pulmonary involvement in ASMD remain to be elucidated. One such aspect concerns the disease‐related factors that might influence the development of severe pulmonary involvement of ASMD.

An emerging technique in pulmonary research is the analysis of exhaled breath [18]. Analysis of exhaled breath with gas chromatography–mass spectrometry (GC–MS) can be used to reveal numerous volatile organic compounds (VOCs) which might be able to serve as biomarkers. Plantier and co‐workers demonstrated the ability to distinguish patients with ILD from healthy controls and even two groups of different ILDs based on VOCs in exhaled breath; they identified 2‐heptanone, 4‐penten‐ol, 2,5‐dimethyl furan, and ethanol as the most important VOCs to distinguish ILD patients from healthy controls [19]. Zanella and co‐workers compared patients with ILD due to systemic sclerosis to healthy controls and identified sixteen VOCs that were able to discriminate patients from healthy subjects, such as cyclohexanone, 6‐methyl‐5‐hepten‐2‐one, and acetophenone [20]. Moreover, research in, for instance, asthma and pulmonary malignancies has identified biomarkers such as acetonitrile, methanol, hexanal, heptanal, octanal, benzaldehyde, undecane, phenylacetaldehyde, decanal, and benzoic acid [21, 22, 23]. These VOCs and pathophysiological processes might be of interest in ASMD as well.

Therefore, we hypothesized exhaled breath analysis might be useful in the identification of biomarkers for lung involvement in ASMD. To investigate this, we conducted an explorative cross‐sectional study in adults with the chronic visceral subtype of ASMD and age‐, sex‐, and smoking habit‐matched healthy controls with the aim to identify markers which might aid in the characterization and/or prediction of the course of pulmonary involvement of ASMD.

## 2 Methods

### 2.1 Participant Recruitment, Screening and Consent

This cross‐sectional explorative study was conducted between December 2022 and December 2023. Patients were recruited at the outpatient clinic for lysosomal storage disorders of the Amsterdam University Medical Center, the national center of expertise for ASMD. In case patients consented to participate in the study, the study visit was combined with the yearly visit to the outpatient clinic. Patients older than 12 years with a confirmed ASMD diagnosis (i.e., decreased acid sphingomyelinase activity and a pathogenic mutation in both alleles of the *SMPD1* gene) were approached to participate in the study. Patients were matched to healthy controls based on age (±5 years), sex, and smoking habit. Healthy controls were screened and were eligible to participate if they were older than 16 years and if they were in general good health as demonstrated by their medical history (specifically no lung disease) and use of medication (especially no inhalation medication). Each patient was matched to at least one healthy control, with a maximum of three since it was demonstrated that the added value of each matched control over 3 is minimal [24].

All participants provided written informed consent for study participation. The study was approved by the local Medical Ethics Committee of the Amsterdam UMC, location Academic Medical Center (2020_298, NL75323.018.20).

### 2.2 Study Visit

All patients underwent general laboratory sampling including hemoglobin measurement as part of their visit; tests were performed at the central laboratory of the hospital. Patients also underwent a high resolution computed tomography (HRCT) scan of the lungs every other year in order to assess signs of pulmonary involvement of ASMD. For patients that did not undergo an HRCT scan at the visit date, the scan one year before the visit date was used. Pulmonary function testing (PFT) was performed by a pulmonary function analyst, and exhaled breath sample collection was performed by EE (collection described below).

For healthy participants the study comprised a singular visit. During this visit they underwent PFT in order to confirm normal lung function as well as exhaled breath sample collection, both performed by EE, who was trained by the pulmonary function analysts of the pulmonary department (for interpretation of PFT, see below). Lastly, 1–2 mL venous blood was drawn in which hemoglobin was measured using an analyzer (ABL 800flex, Radiometer, Zoetermeer, the Netherlands).

To mitigate potential seasonal variations in sample composition, samples from healthy participants were obtained within a month of the corresponding patient's visit.

### 2.3 Definition of Pulmonary Involvement

Patients were considered to have pulmonary involvement of ASMD when DLCO was < 80% of predicted or when ground glass opacities or fibrosis were present on HRCT. Signs of fibrosis were traction bronchiectasis, volume loss, and honeycombing. The ASMD patients with pulmonary involvement and their matched controls formed a subgroup which was used in several analyses.

### 2.4 Pulmonary Function Testing

PFT results of all participants were adjusted based on the guidelines of the Global Lung Function Initiative [25, 26]. PFT included measurement of forced vital capacity (FVC) in liters, forced expiratory volume in 1 s (FEV_1_) in liters, and diffusion capacity of the lungs for carbon monoxide (DLCO) in mmol/min*kPa corrected for hemoglobin levels.

### 2.5 HRCT Scores

HRCT scans were assessed by two investigators (EE and LM). The scoring system for HRCT of the lungs specific for ASMD as proposed by Mendelson et al. was used [9]. Images were obtained at four levels: (I) the upper lung zone corresponding to the aortic arch, (II) the midlung zone corresponding to the carina, (III) the lower lung zone midway between the carina and the higher hemidiaphragm, and (IV) also the lower lung zone, corresponding to 1 cm above the higher hemidiaphragm. The scores of the two lower lung zones (III and IV) were averaged to take into account that the lowest lung zone just above the diaphragm is commonly the first and most severely affected area. Each level for both lungs was scored on (a) the presence of interstitial lung disease (score 0 or 1), (b) the presence of thickened interlobular septa and intralobular lines (score 0 or 1), and (c) the presence of ground glass opacities (GGO) on a scale of 0–3 as follows: no GGO was scored at 0, 0%–25% GGO was scored at 1, 26%–50% GGO was scored at 2, and more than 50% GGO was scored at 3. To quantify GGO, Syngo.via CT Pneumonia software analysis program (Siemens Healthineers) was used. HRCT score is the sum of the scores for both lungs of the upper lung zone, the midlung zone, and the average of the two lower lung zones and can range from 0 to a maximum of 30.

### 2.6 Measuring and Definitions of Clinical Markers of ASMD

For patients, several biochemical plasma markers were measured as part of their yearly visit: chitotriosidase activity, chemokine C‐C motif ligand 18 (CCL18), lysosphingomyelin (LSM) and N‐palmitoyl‐phosphocholineserine (PPCS, also known as lysosphingomyelin‐509). These markers were measured as described earlier [27, 28, 29]. *CHIT1* phenotypes were determined, and in case of heterozygosity for the 24‐bp duplication in the *CHIT1* gene, chitotriosidase activity was multiplied by two [30]. In case of homozygosity, patients were chitotriosidase deficient and excluded from the relevant analyses.

Some clinical parameters were collected from their medical data in order to define splenic and hepatic involvement (i.e., spleen volume, platelet levels, liver volume and ALT levels). Thrombocytopenia was defined as platelet levels < 100 × 10^9^/l. Reference values of alanine aminotransaminase (ALT) levels are gender‐specific; AST levels are elevated when > 45 U/L for males or > 34 U/L for females. Data on hepatosplenomegaly were acquired through imaging techniques (preferably magnetic resonance imaging (MRI), in some cases ultrasound (e.g., patients with claustrophobia)). Precise measurements of spleen and liver volumes were feasible only with MRI. Splenomegaly and hepatomegaly were defined based on previous definitions with volume measured on magnetic resonance imaging (MRI) adjusted to body weight into multiples of normal (MN) [31]. In cases where ultrasound was employed, the report indicated the presence of splenomegaly or hepatomegaly.

### 2.7 Exhaled Breath Sample Collection

Two types of exhaled breath samples were collected for all participants: one single breath to measure volatile organic compounds (VOCs) with gas chromatography–mass spectrometry (GC–MS) and exhaled breath condensate to obtain samples for metabolomics analysis.

Participants were asked to refrain from eating and drinking two hours prior to sample collection [32, 33]. Breath samples for GC–MS analysis were obtained as previously described [34]. In short, participants breathed through a two‐way non‐rebreathing valve with an inspiratory carbon filter (A1, Honeywell North Safety Products, Middelburg, NL) to filter inhaled air for ten breaths. After ten breaths, they inhaled deeply, and before they exhaled, a sample bag was attached to the valve, enabling the collection of one exhaled breath. Within 30 min, a thermal desorption tube (Tenax GR 60/80, Camsco, Houston, TX, USA) was used to store the VOCs. The tube was sampled with 500 mL of breath at a flow of 250 mL/min with a diaphragm suction pump (GSP‐300FT‐2 AirSamplingPump, Gastec Corporation, Ayase, Japan). Tubes were stored at 4°C and were analyzed with GC–MS in the same batch.

Exhaled breath condensate (EBC) was obtained using an EBC collector (TURBO 14, MEDIVAC, Parma, Italy). Participants wore a nose clip and breathed at tidal volume during fifteen minutes. The exhaled breath was cooled down to 4°C, resulting in several milliliters of breath condensate. Samples were stored at −80°C and metabolomics analysis was done in one batch. Exhaled breath condensate was not collected from one patient due to logistic restraints the day of the visit.

### 2.8 GC–MS Analysis, Data Processing and Compound Identification

Analysis with GC–MS was performed as previously described [34]. For analysis by GC–MS, the thermal desorption tubes were placed into a thermal desorption unit (Markes TD100 Cincinnati, Ohio, United states of America) and heated to 280°C in 5 min with a flow of 30 mL/min. This caused the VOCs to release from the thermal desorption tubes; subsequently, they were captured on a cold trap at 10°C. This cold trap was heated rapidly to 300°C for one minute, after which the VOCs were splitless‐injected through a transfer line at 180°C onto an Inertcap 5MS/Sil gas‐chromatography column (30 m, ID 175 0.25 mm, film thickness 1 um, 1,4‐bis(dimethylsiloxy)phenylene dimethyl‐polysiloxane, Restek, Breda, The Netherlands) at 1.2 mL/min. Next, the VOCs were ionized using electron ionization (70 eV) and the fragment ions were detected using a quadrupole mass spectrometer (GCMS‐GP2010, Shimadzu, Den Bosch, The Netherlands) with a scan range of 37–300 Da.

GC–MS data were pre‐processed by performing denoising and peak detection, and alignment as previously described [21] using the XCMS package (Scripps Center for Metabolomics, La Jolla, CA) in Rstudio (v4.3.2) [35]. This resulted in an ion fragment peak table for further analysis.

The National Institute of Standards and Technology (NIST) library was used to identify compounds based on retention time and weight. Only VOCs below 200 g/mol were assessed, since these are considered volatile at room temperature. In some cases, several retaining fragments (i.e., molecule fragments) could be attributed to one molecule; in that case, retaining fragments were called after the molecule and a Roman numeral was added (for instance nine retaining fragments could be attributed to acetic acid, these were called acetic acid I to IX).

### 2.9 Metabolomics Analysis and Metabolite Identification

Metabolomics analysis of exhaled breath condensate samples was performed as previously described, with minor adjustments [36]. The internal standard is described in the supplemental methods. Solvents were added to achieve a total volume of 500 μL methanol, 500 μL water, and 1 mL chloroform. After thorough mixing, samples were centrifuged for 10 min at 14.000 rpm. The polar top layer was transferred to a new 1.5 mL tube and dried using a vacuum concentrator at 60°C. Dried samples were reconstituted in 100 μL 6:4 (v/v) methanol: water. Metabolites were analyzed using a Waters Acquity ultra‐high performance liquid chromatography system coupled to a Bruker Impact II Ultra‐High Resolution Qq‐Time‐Of‐Flight mass spectrometer. Samples were kept at 12°C during analysis, and 5 μL of each sample was injected. Chromatographic separation was achieved using a Merck Millipore SeQuant ZIC‐cHILIC column (PEEK 100 × 2.1 mm, 3 μm particle size). Column temperature was held at 30° *C.* The *mobile* phase consisted of (A) 1:9 (v/v) acetonitrile: water and (B) 9:1 (v/v) acetonitrile: water, both containing 5 mmol/L ammonium acetate. Using a flow rate of 0.25 mL/min, the LC gradient consisted of: Dwell at 100% Solvent B, 0–2 min; Ramp to 54% Solvent B at 13.5 min; Ramp to 0% Solvent B at 13.51 min; Dwell at 0% Solvent B, 13.51–19 min; Ramp to 100% B at 19.01 min; Dwell at 100% Solvent B, 19.01–19.5 min. The column was equilibrated by increasing the flow rate to 0.4 mL/min at 100% B for 19.5–21 min. MS data were acquired using negative and positive ionization in full scan mode over the range of m/z 50–1200. Data were analyzed using Bruker TASQ software version 2021.1.2.452. All reported metabolite intensities were normalized to internal standards with comparable retention times and responses in the MS. Metabolite identification has been based on a combination of accurate mass and (relative) retention times, compared to the analysis of a library of standards.

### 2.10 Statistical Analysis

Statistical analyses were performed in Rstudio (v4.3.2). Demographic data were shown in median and ranges, unless stated otherwise. Differences between groups were calculated using the Mann–Whitney U test or Fisher's exact test for continuous or dichotomous data respectively. For both omics layers, exhaled VOCs and EBC, compounds of interest were selected based on a *p*‐value < 0.05 and 2log fold change < −0.58 or > 0.58 (which corresponds with a fold change of 0.67 or of 1.5, respectively) when comparing patients to healthy controls. The 2log fold change cut off value of < −0.58 or > 0.58 has been used in large data sets to reflect a moderate change between two compounds [37, 38]. Spearman's rank correlation coefficient was calculated to investigate correlations, reciprocal for the compounds of interest as well as between the compounds of interest and clinical markers (i.e., PFT for all participants as well as pulmonary imaging and biochemical markers for patients). Correction for multiple testing was not performed due to the explorative nature of this study, in order to avoid type II errors. Sparse Partial Least Squares‐Discriminant Analysis (sPLSDA) in combination with Area Under the Receiver Operating Characteristic Curve (AUROCC, mixOmics [39]) were applied to test the discriminative power of a GC–MS and metabolomics combined dataset. Furthermore, a logistic regression was performed to identify the most discriminating compounds of interest.

## 3 Results

**FIGURE 1:**
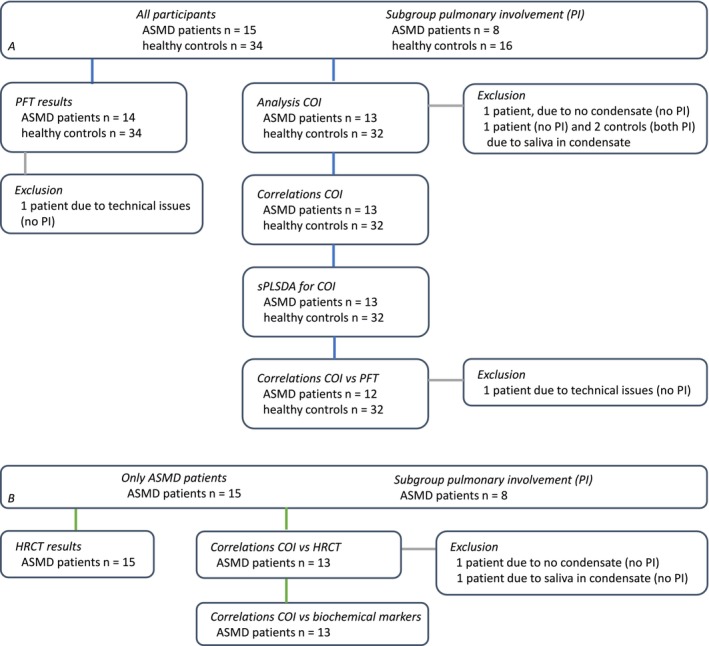
Overview of analysis and data available per analysis. (A) Data available for ASMD patients and healthy controls, (B) data available for ASMD patients. ASMD, acid sphingomyelinase deficiency; COI, compounds of interest; HRCT, high‐resolution computed tomography; PFT, pulmonary function testing; PI, pulmonary involvement; sPLSDA, sparse partial least squares‐discriminant analysis.

In total, 49 participants were included in the study: fifteen adult, chronic visceral ASMD patients and 34 age‐, sex‐, and smoking habit‐matched healthy controls. Each patient had at least one healthy participant matched to them. Of the nineteen eligible patients monitored at the outpatient clinic for lysosomal storage disorders of the Amsterdam University Medical Center, fifteen were willing to participate, resulting in a recruitment success rate of 79%. Healthy participants had no prior lung disease and used no inhalation medication. For 13 patients, the sample collection of all matched healthy participants took place within one month. Three healthy participants matched to two patients, respectively, were seen two, two, and three months apart from the patients' visit date; this was due to practical issues. All healthy controls and eight patients provided the samples in the pulmonary function department. Due to logistics on their annual visit day, five patients provided samples in the outpatient clinic and two patients in the radiology department. PFT results were available for all participants except for one patient. HRCT scans were available for all patients; for one of them, only a HRCT scan performed the year after the study visit was available and therefore assessed. Characteristics of patients and healthy controls and the subgroup of patients with pulmonary involvement and their matched controls are depicted in Table 1. The analyses and the data available per analysis are depicted in Figure 1.

**TABLE 1:** Demographics.

	ASMD patients	Healthy controls	*p*‐value	ASMD patients with pulmonary involvement	Matched healthy controls	*p*‐value
Amount	15	34		8	16	
Demography						
Age (years)	33 (19‐67)	29.5 (18‐69)	0.610	32 (19‐67)	29 (18‐69)	0.690
Sex (male)	10 (67%)	21 (62%)	1.000	5 (63%)	9 (56%)	1.000
Smoking	5 (33%)	10 (29%)	0.735	3 (38%)	5 (31%)	1.000
Pulmonary function testing						
FVC	100 (74‐140)	114 (97‐159)	0.003	95 (74‐140)	112 (97‐159)	0.050
FEV1	89 (60‐118)	112 (90‐160)	< 0.001	86 (60‐118)	109 (93‐160)	0.011
DLCO	78 (28‐109)	108 (80‐139)	< 0.001	74 (28‐78)	97 (80‐131)	< 0.001
Pulmonary imaging						
HRCT score	12 (0‐24)			14 (12‐24)		
ASMD manifestations						
Splenectomy	2 (13%)			1 (13%)		
Splenomegaly	12 (100%)^a^			7 (100%)		
Thrombocytopenia	0 (0%)			0 (0%)		
Hepatomegaly	8 (67%)^a^			3 (38%)		
Elevated ALT	7 (47%)			3 (38%)		
Chitotriosidase activity (nmol/ml.hr)	928 (256‐6783)^b^			1342 (302‐6783)^d^		
LSM (nmol/l)	109 (14‐249)			114 (55‐2449)		
PPCS (nmol/l)	1292 (227‐3257)			1413 (435‐3257)		
CCL18 (ng/ml)	533 (115‐1240)^c^			538 (322‐1240)^d^		

*Note:* Values are depicted in median and ranges or number of patients with percent of patients where appropriate, *p*‐values were calculated with Mann‐Whitney U test or Fisher's exact test where appropriate.

Abbreviations: ALT, alanine aminotransaminase; CCL18, chemokine C‐C motif ligand 18; DLCO, diffusion capacity of the lungs for carbon monoxide; FEV1, forced expiratory volume in 1 second; FVC, forced vital capacity; HRCT, high resolution computed tomography; LSM, lysosphingomyelin; PPCS, N‐palmitoyl‐phosphocholineserine.

^a^ Data available for 12 patients.

^b^ Data available for 13 patients.

^c^ Data available for 14 patients.

^d^ Data available for 7 patients.

**FIGURE 2:**
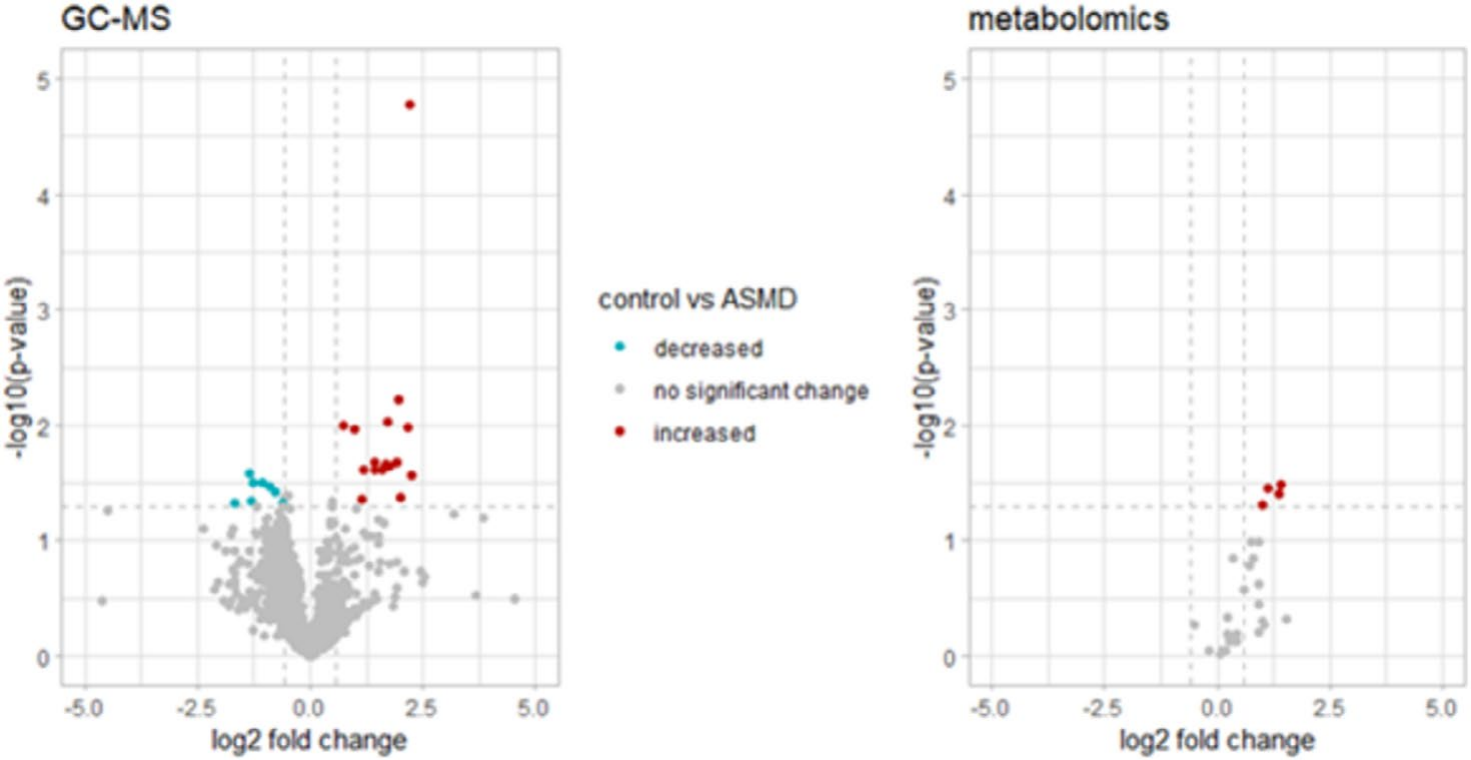
Compounds of interest derived from GC‐MS and metabolomics analyses summarized in a table and volcano plots. *Table*: Retention time is only provided for GC‐MS compounds. For compounds composed of multiple molecular fragments the largest 2log fold change and corresponding *p*‐value are depicted. *Volcano plots*: All compounds are represented by a dot. The x‐axis displays the log 2 fold change of the compounds of healthy controls as compared to ASMD patients. The y‐axis displays the *p*‐value with a −log10 transformation. The dotted lines represent the cut‐off values for the compounds of interest. The two vertical lines represent a fold change of 0.67 or 1.5 (which corresponds to a log 2 fold change of −0.58 or 0.58). The horizontal line represents a *p*‐value of 0.05 (which corresponds to a −log10 (*p*‐value) of 1.3). The compounds of interest are depicted in blue (decreased in healthy controls as compared to ASMD patients) and red (increased in healthy controls as compared to ASMD patients). GC‐MS, gas chromatography‐mass spectrometry.

**FIGURE 3:**
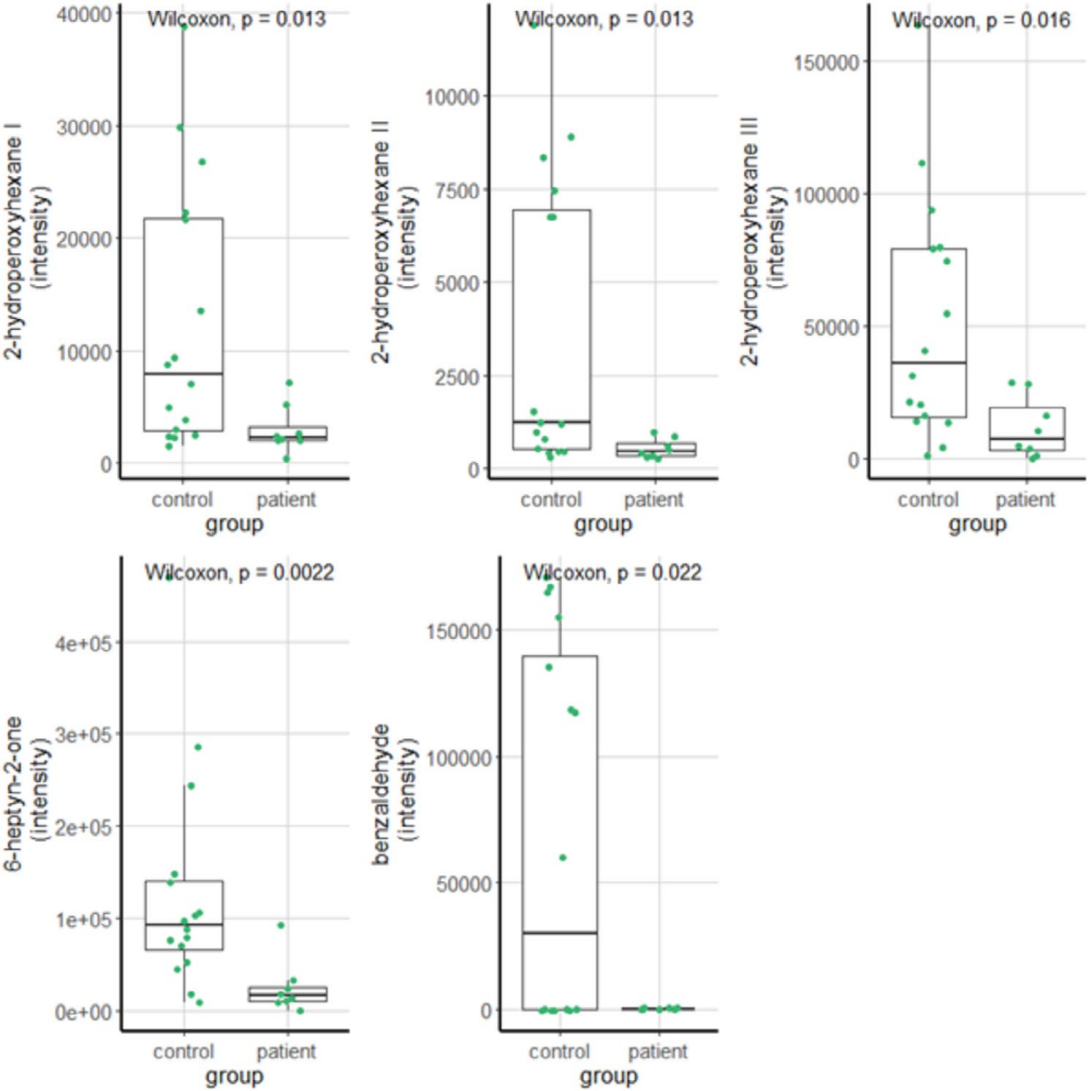
Boxplots comparing patients with pulmonary involvement to their matched controls for several compounds of interest. For 2‐hydroperoxyhexane, three different molecule fragments were measured (I, II and III).

To identify the VOC‐ and EBC‐based compounds of interest, exhaled breath data of all patients were compared to all controls. Four participants were excluded: one patient whose exhaled breath condensate was not obtained due to logistic reasons, one patient whose exhaled breath condensate contained saliva, and two healthy controls with saliva in the exhaled breath condensates as well. After these exclusions, there was still at least one matched healthy control per patient. The VOCs and metabolites that met the predefined criteria formed the compounds of interest and were summarized and depicted in Figure 2. The GC–MS analysis of the exhaled breath samples resulted in 24 retaining fragments originating from eleven VOCs. These eleven compounds of interest all had a carbon base of seven to twelve carbon atoms except for one (acetic acid, with a backbone of two carbon atoms). Seven compounds had a simple carbon base without double bonds (e.g., alkanes such as decane and dodecane and carbon chains with functional groups such as 2‐hydroperoxyhexane and 2‐methylnonane). One compound of interest had a double bond (i.e., 4‐pentenyl acetate) and one had a triple bond (i.e., 6‐heptyn‐2‐one). Two compounds contained a benzene ring (i.e., benzaldehyde and acetophenone). The metabolomics analysis yielded four more compounds of interest; all were amino acids (Figure 2). When analyzing these compounds of interest in the subgroup of patients with pulmonary involvement and their matched controls, significant differences were found for 2‐hydroperoxyhexane I t/m III (respectively *p* = 0.016, p = 0.016, *p* = 0.018), 6‐heptyn‐2‐one (*p* = 0.004) and benzaldehyde (*p* = 0.021, see Figure 3).

Correlations between the compounds of interest were examined in a correlation matrix (depicted in Table 2). All molecular fragments measured with GC–MS, which were allocated to the same molecule, showed strong mutual correlation (for instance the nine different compounds that were identified to belong to acetic acid), implying a correct identification.

**TABLE 2:** Correlation plot of compounds of interest.

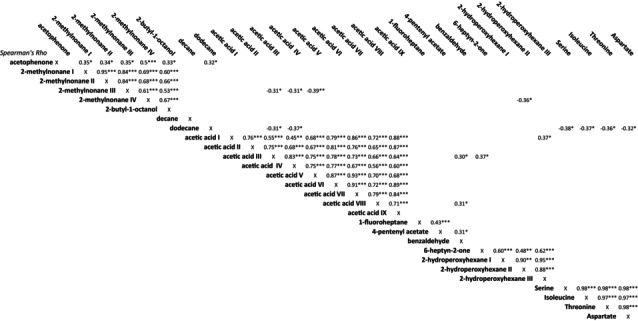

*Note:* Only significant correlations are shown, *p < 0.05, **p < 0.01, ***p < 0.001.

For all participants of whom both exhaled breath samples were available, correlations between the compounds of interest and PFT results were tested. DLCO correlated with four compounds of interest: 2‐hydroperoxyhexane I to III (respectively *R* = 0.55, *p* < 0.001, *R* = 0.58, *p* < 0.001, *R* = 0.56, *p* < 0.001), 2‐methylnonane IV (R = −0.41, *p* = 0.006), 2‐butyl‐1‐octanol (R = −0.32, *p* = 0.036) and 6‐heptyn‐2‐one (*R* = 0.46, *p* = 0.002). In the subgroup of patients with pulmonary involvement and their matched controls, correlations between DLCO and 2‐hydroperoxyhexane I to III (respectively *R* = 0.58, *p* = 0.003, *R* = 0.68, *p* < 0.001, R = 0.56, *p* = 0.004) and 6‐heptyn‐2‐one (*R* = 0.49, *p* = 0.015) were also found. An additional correlation between DLCO and benzaldehyde was found in this subgroup (*R* = 0.45, *p* = 0.026). In Supplemental Table 1, the significant correlations between the compounds of interest and PFT results are summarized.

**FIGURE 4:**
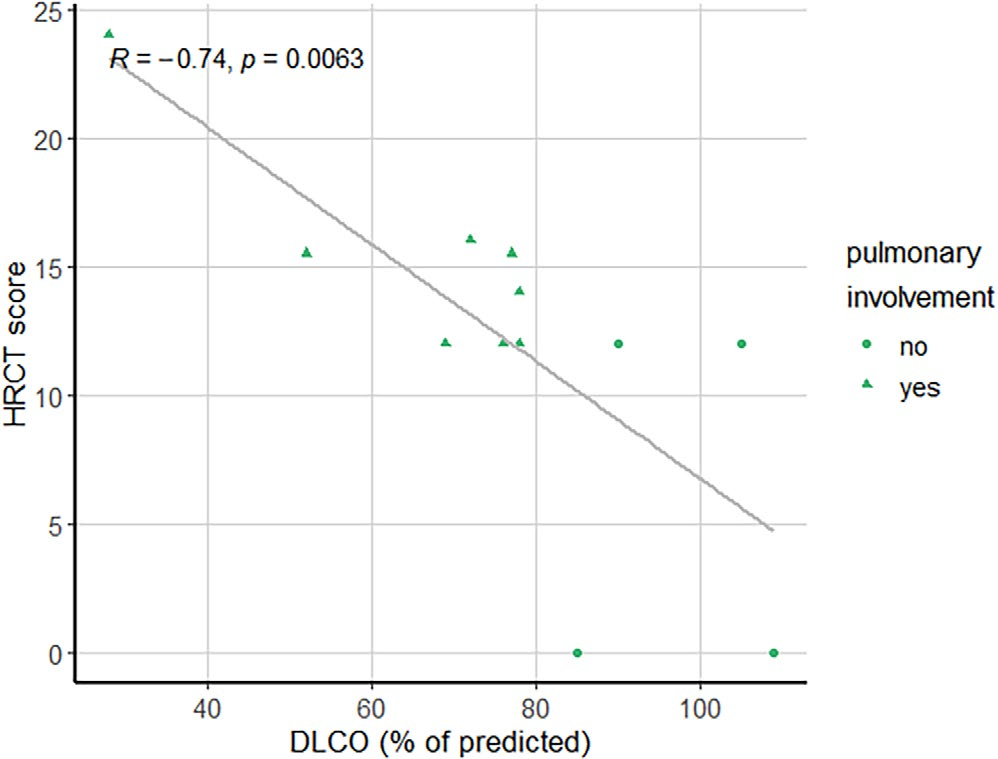
Correlation between DLCO and HRCT score. DLCO, diffusion capacity of the lungs for carbon monoxide; HRCT, high resolution computed tomography.

None of the patients showed signs of pulmonary fibrosis on HRCT scan of the lungs. Median HRCT score was 12 (range 0–24). HRCT score correlated with DLCO (R = −0.74, *p* = 0.006, see Figure 4). Furthermore, correlations were investigated between compounds of interest and HRCT score of patients. Three compounds showed significant correlations with HRCT score: 2‐hydroperoxyhexane II, serine, and threonine (respectively R = −0.70, *p* = 0.007, R = −0.57, *p* = 0.042, R = −0.48, *p* = 0.033). One compound of interest showed a correlation with HRCT score in the subgroup of patients with pulmonary involvement and their matches, which was aspartate (R = −0.76, *p* = 0.028).

Correlations between compounds of interest and biochemical markers of ASMD were tested. This yielded four significant correlations; three compounds of interest correlated with chitotriosidase activity (i.e., 4‐pentenyl acetate, R = −0.96, *p* < 0.001, 1‐fluoroheptane, R = −0.63, *p* = 0.037 and 2‐butyl‐1‐octanol, R = −0.62, *p* = 0.048) and one compound of interest correlated with LSM (i.e., 4‐pentenyl acetate, R = −0.62, *p* = 0.024). In the subgroup of patients with pulmonary involvement and their matched controls, correlations were found between chitotriosidase activity and 1‐fluoroheptane (R = −0.78, *p* = 0.041) and 4‐pentenyl acetate (*R* − 0.95, *p* < 0.001) and for CCL18 and 2‐hydroperoxyhexane I and III (respectively R = −0.78, *p* = 0.048 and R = −0.86, *p* = 0.024). For all analyses in which only patients were included, testing for confounders (i.e., age, sex, smoking and the season in which the samples were collected) was performed and yielded no confounding effects.

**FIGURE 5:**
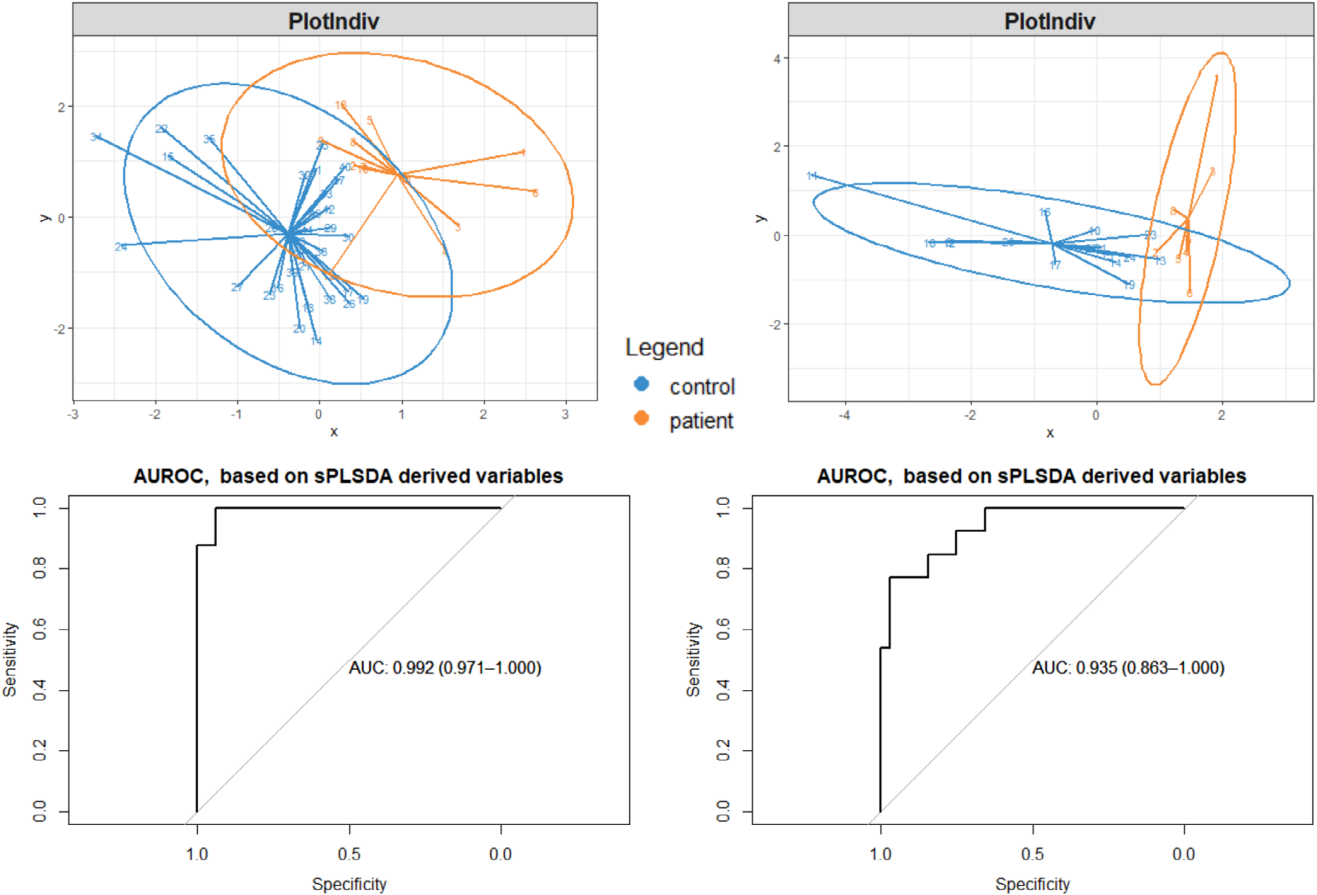
The sPLSDA and AUROCC analyses using compounds of interest. Depicted for all participants (left) and the subgroup of patients with pulmonary involvement and their matched controls (right). Individual participants are represented by numbers; circles represent the 95% confidence interval. AUROCC, area under the receiver operating characteristic curve; sPLSDA, sparse partial least squares‐discriminant analysis.

Lastly, to investigate the ability to distinguish patients from healthy controls, sPLSDA and AUROCC analyses were performed using the compounds of interest (see Figure 5). In the sPLSDA for all participants, the groups showed overlap and sPLSDA components 1 and 2 captured 17% of the variance (comp 1: 9%, comp 2: 8%). The AUROCC for the sPLSDA was 0.94 (95% confidence interval 0.863–1.000). The model identified 6‐heptyn‐2‐one, decane, benzaldehyde, 4‐pentenylacetate, acetophenone, 1‐fluoroheptane, serine, and isoleucine as the most discriminating compounds of interest. In the sPLSDA for the subgroup of patients with pulmonary involvement and their matched controls, less overlap was observed, and the two X‐sPLSDA components accounted for 26% of the variance (comp 1: 21%, comp 2: 5%). The AUROCC was 0.99 (95% confidence interval 0.971‐1.000). The model identified benzaldehyde, 6‐heptyn‐2‐one, 2‐hydroperoxyhexane, and decane as the most contributing compounds of interest. In conclusion, the sPLSDA model was better able to distinguish patients with pulmonary involvement from their matched controls, although an overfit is likely. Logistic regression analysis showed similar outcomes and did not have added value.

## 4 Discussion

Exhaled breath samples of 15 ASMD patients and 34 matched controls were analyzed, and based on significant differences between the two groups, fifteen VOC‐ and EBC‐based compounds of interest were identified. In Supplemental Table 2, all analyses and the compounds of interest that differed significantly or showed a correlation are summarized for both all participants as well as for the subgroup of patients with pulmonary involvement and their matched controls. The most promising compounds of interest were 2‐hydroperoxyhexane and 6‐heptyn‐2‐one because they differed significantly for both all participants as well as the subgroup of patients with pulmonary involvement and their matched controls, and they showed correlations with PFT results, especially 2‐hydroperoxyhexane, which also correlated with HRCT score (see Supplemental Table 2). Moreover, 4‐pentenyl acetate correlated with chitotriosidase activity in both groups and with LSM for all participants. In an sPLSDA and AUROCC analysis, the model was better able to distinguish the patients with pulmonary involvement from their matched controls than all patients from all controls.

No references to these most promising compounds of interest or their synonyms were identified in any studies related to pulmonary disorders. Therefore, they might be ASMD‐specific and could have a role in diagnosing the disease or monitoring disease progression. In fact, no previous research studying exhaled breath samples with GC–MS and metabolomics analyses in ASMD has been conducted. As mentioned in the introduction, these techniques have been applied in pulmonary disorders such as ILD and lung cancer [18, 19, 21, 22, 23]. Four of the fifteen compounds of interest identified in this study were previously suggested as markers of several pulmonary diseases in exhaled breath: benzaldehyde, acetophenone, decane, and dodecane. Benzaldehyde was identified as a VOC specific for lung cancer in multiple studies comparing exhaled breath of lung cancer patients to that of healthy controls [23, 40, 41]. Acetophenone was suggested as a marker for ILD caused by systemic sclerosis in a study comparing systemic sclerosis patients to healthy controls [20]. Decane and dodecane were found to be potential biomarkers of subclinical pulmonary oxygen toxicity [42]. Dodecane also emerged as a marker for lung cancer in exhaled breath samples of patients and in vitro samples of several lung tumors [43, 44].

Since little is known about the specific compounds of interest in the context of ASMD or pulmonary disease, it was of interest to assess the biochemical groups they belong to. Alkanes, methylalkanes, and hydroperoxides, such as decane, dodecane, 2‐methylnonane, and 2‐hydroperoxyhexane, are believed to be (intermediate) products of lipid peroxidation (for instance derived from fatty acids) resulting from cell membranes due to oxidative stress [42, 45, 46]. Lipid peroxidation has been described as a pathophysiological process in several lysosomal storage diseases; therefore, these markers might be a reflection of this process transpiring in the lungs [47, 48, 49, 50]. Moreover, aberrant amino acid profiles have been described to potentially be associated with inflammatory processes and oxidative stress in chronic obstructive pulmonary disease (COPD) [51, 52, 53]. Sphingolipids were also increased in the plasma and sputum of COPD patients; the hypothesis was raised that the inhalation of respiratory irritants causes an increase of neutral sphingomyelinase, which in turn converts sphingomyelin into ceramide [53, 54, 55]. In ASMD, the accumulation of sphingomyelin might lead to increased local release of ceramide and similar pathophysiology without the presence of respiratory irritants. Lastly, the metabolism of sphingolipids is disrupted in several types of pulmonary cancer [56, 57, 58]. In Gaucher disease (OMIM 230800), a lysosomal storage disease similar to ASMD, the accumulation of sphingolipids is associated with an increased risk for several types of cancer, such as hepatocellular carcinoma, lung cancer, and myeloma [59]. This raises the hypothesis that similar pathophysiological processes might be involved in both ASMD and several types of (pulmonary) cancer.

The HRCT score applied in this study could have value in the monitoring of ASMD patients. The current guideline advises pulmonary imaging at least every 2–4 years, and HRCT is currently the optimal imaging technique available [60]. A quantitative score, as used in this study, enables a comparison between multiple scans performed during follow‐up in contrast to qualitative reports by a radiologist. We assessed multiple HRCT scans of ten patients from this study, and HRCT scores were predominantly stable over the course of three to eighteen years, with a maximum increase of 3.5 points (over four years). The HRCT scores of the patients in this cohort correlated strongly with DLCO measurements. Mendelson et al., who suggested the HRCT score used in this study, reported a weak correlation between the HRCT score and PFT [9]. No other studies compared pulmonary imaging to PFT to the best of our knowledge.

This study has some limitations. Firstly, the amount of patients who participated in the study is small. However, ASMD is a rare disease and all patients monitored in our center were invited to participate, and by matching all patients to one to three healthy controls, the study population was optimized. We also limited the matching criteria to age, sex, and smoking status for feasibility; other metabolic conditions such as diabetes or cardiovascular disease might have an influence on exhaled breath, which was not accounted for in the matching process. Secondly, no correction for multiple testing was performed in order to avoid type II errors. Future studies in larger populations should ideally include a validation cohort in order to overcome type I errors, which occur as a result of multiple testing. Lastly, we did not find any lipids or lipid derivatives in the exhaled breath condensate of the ASMD patients. In a previous study, the presence of several lysophosphatidic acids in exhaled breath condensate of patients with idiopathic pulmonary fibrosis is described [61]. However, these lipids have a molecular weight > 200 g/mol and are therefore most likely originating from aerosols, which we aimed not to collect in this study.

In conclusion, we identified several VOCs and EBC‐based compounds of interest in the exhaled breath of ASMD patients which distinguish them from healthy controls. The most promising markers based on our analyses (i.e., 2‐hydroperoxyhexane and 6‐heptyn‐2‐one) have not been described in previous studies in the pulmonary field and might be ASMD‐specific. Other compounds of interest have been described in the context of systemic sclerosis (i.e., acetophenone) or lung cancer (i.e., benzaldehyde and dodecane). Some markers were associated with the pathophysiological process of lipid peroxidation (i.e., decane, dodecane and 2‐methylnonane). Furthermore, using the HRCT score, pulmonary involvement as observed on pulmonary imaging could be quantified, and a correlation with DLCO was established. This study could serve as a basis for future studies, in which markers could be measured in the exhaled breath of a larger cohort with ASMD patients with more severe pulmonary involvement in order to further investigate the ability of the potential markers to distinguish ASMD patients with pulmonary involvement from patients without or ASMD patients from healthy controls, to assess the severity of pulmonary involvement of ASMD, or to evaluate the effect of therapy.

## Consent

All procedures followed were in accordance with the ethical standards of the responsible committee on human experimentation (institutional and national) and with the Helsinki Declaration of 1975, as revised in 2000. Informed consent was obtained from all patients for being included in the study.

## Conflicts of Interest

Bauke V. Schomakers, Michel van Weeghel, Suzanne W.J. Terheggen‐Lagro, Lilian J. Meijboom, Paul Brinkman, and Barbara Sjouke have no competing interests to declare. Eline C.B. Eskes and Carla E.M. Hollak were involved in a pre‐marketing study with Sanofi Genzyme.

## Supporting information


**Data S1.** Supporting Information.

**Supplemental Table 1.** Correlations between PFT results and compounds of interest. Correlations were calculated for all participants and for the subgroup of ASMD patients with pulmonary involvement and their matched controls. Spearman’s rho is shown only for significant correlations. *p < 0.05, **p < 0.01, ***p < 0.001. DLCO, diffusion capacity of the lungs for carbon monoxide; FEV1, forced expiratory volume in 1 second; FVC, forced vital capacity.
**Supplemental Table 2**. Summary of comparisons and correlations from this study for all participants and the subgroup of patients with pulmonary involvement and their matched controls. Depicted are the compounds of interest that differed significantly between patients and controls (first line COI analysis) and the compounds of interest for which a significant correlation was found (all other lines). CCL18, chemokine C‐C motif ligand 18; COI, compounds of interest; DLCO, diffusion capacity of the lungs for carbon monoxide; FEV1, forced expiratory volume in 1 second; FVC, forced vital capacity; LSM, lysosphingomyelin; sPLSDA, sparse partial least squares‐discriminant analysis.
