# Supplementary material for: Exploring Exhaled Breath Analysis in Adults With Chronic Visceral Acid Sphingomyelinase Deficiency to Identify Potential Biomarkers of Pulmonary Involvement
Source: J Inherit Metab Dis. 2025 Jul 27;48(4):e70039. doi: 10.1002/jimd.70039 (PMC12301289; doi:10.1002/jimd.70039)
Supplement: Supplementary file 1 — Data S1. Supporting Information. [file JIMD-48-0-s002.docx]

**Supplemental methods**

*Internal standard metabolomics analysis*

In a 2 mL tube, the following amounts of internal standard dissolved in water were added to each sample of 400 µL of breath condensate: adenosine-15N5-monophosphate (5 nmol), adenosine-15N5-triphosphate (5 nmol), D4-alanine (0.5 nmol), D7-arginine (0.5 nmol), D3-aspartic acid (0.5 nmol), D3-carnitine (0.5 nmol), D4-citric acid (0.5 nmol), 13C1-citrulline (0.5 nmol), 13C6-fructose-1,6-diphosphate (1 nmol), 13C2-glycine (5 nmol), guanosine-15N5-monophosphate (5 nmol), guanosine-15N5-triphosphate (5 nmol), 13C6-glucose (10 nmol), 13C6-glucose-6-phosphate (1 nmol), D3-glutamic acid (0.5 nmol), D5-glutamine (0.5 nmol), D5-glutathione (1 nmol), 13C6-isoleucine (0.5 nmol), D3-lactic acid (1 nmol), D3-leucine (0.5 nmol), D4-lysine (0.5 nmol), D3-methionine (0.5 nmol), D6-ornithine (0.5 nmol), D5-phenylalanine (0.5 nmol), D7-proline (0.5 nmol), 13C3-pyruvate (0.5 nmol), D3-serine (0.5 nmol), D6-succinic acid (0.5 nmol), D4-thymine (1 nmol), D5-tryptophan (0.5 nmol), D4-tyrosine (0.5 nmol) and D8-valine (0.5 nmol).
