# Supplementary material for: Exploring Exhaled Breath Analysis in Adults With Chronic Visceral Acid Sphingomyelinase Deficiency to Identify Potential Biomarkers of Pulmonary Involvement
Source: J Inherit Metab Dis. 2025 Jul 27;48(4):e70039. doi: 10.1002/jimd.70039 (PMC12301289; doi:10.1002/jimd.70039)
Supplement: Supplementary file 2 — Supplemental Table 1. Correlations between PFT results and compounds of interest. Correlations were calculated for all participants and for the subgroup of ASMD patients with pulmonary involvement and their matched controls. Spearman’s rho is shown only for significant correlations. *p < 0.05, **p < 0.01, ***p < 0.001. DLCO, diffusion capacity of the lungs for carbon monoxide; FEV1, forced expiratory volume in 1 second; FVC, forced vital capacity. Supplemental Table 2. Summary of comparisons and correlations from this study for all participants and the subgroup of patients with pulmonary involvement and their matched controls. Depicted are the compounds of interest that differed significantly between patients and controls (first line COI analysis) and the compounds of interest for which a significant correlation was found (all other lines). CCL18, chemokine C‐C motif ligand 18; COI, compounds of interest; DLCO, diffusion capacity of the lungs for carbon monoxide; FEV1, forced expiratory volume in 1 second; FVC, forced vital capacity; LSM, lysosphingomyelin; sPLSDA, sparse partial least squares‐discriminant analysis. [file JIMD-48-0-s001.docx]

|  | **All patients vs all controls** | | | **Patients with pulmonary involvement vs matched controls** | | |
| --- | --- | --- | --- | --- | --- | --- |
| *Spearman's Rho* | **DLCO** | **FEV1** | **FVC** | **DLCO** | **FEV1** | **FVC** |
| **2-hydroperoxyhexane_I** | 0.55*** | 0.36* | 0.33* | 0.58** | 0.45* | *NS* |
| **2-hydroperoxyhexane_II** | 0.58*** | *NS* | *NS* | 0.68*** | *NS* | *NS* |
| **2-hydroperoxyhexane_III** | 0.56*** | 0.36* | 0.34* | 0.56** | *NS* | *NS* |
| **2-methylnonane_IV** | -0.41** | -0.31* | *NS* | *NS* | *NS* | *NS* |
| **2-butyl-1-octanol** | -0.32* | *NS* | *NS* | *NS* | *NS* | *NS* |
| **6-heptyn-2-one** | 0.46** | *NS* | *NS* | 0.49* | *NS* | *NS* |
| **acetophenone** | *NS* | -0.31* | *NS* | *NS* | *NS* | *NS* |
| **benzaldehyde** | *NS* | *NS* | *NS* | 0.45* | *NS* | *NS* |
| **acetic acid II** | *NS* | *NS* | *NS* | *NS* | 0.43* | *NS* |
| **acetic acid VIII** | *NS* | *NS* | *NS* | *NS* | 0.44* | *NS* |
| **acetic acid IX** | *NS* | *NS* | *NS* | NS | 0.47* | *NS* |

**Supplemental table 1 Correlations between PFT results and compounds of interest.** Correlations were calculated for all participants and for the subgroup of ASMD patients with pulmonary involvement and their matched controls. Spearman’s rho is shown only for significant correlations. * p<0.05, ** p<0.01, *** p <0.001. DLCO: diffusion capacity of the lungs for carbon monoxide, FEV1: forced expiratory volume in 1 second, FVC: forced vital capacity.

| Comparison/correlation | All participants | Patients with pulmonary involvement and their matched controls |
| --- | --- | --- |
| COI | fifteen compounds of interest, see figure 3 | 2-hydroperoxyhexane, 6-heptyn-2-one, benzaldehyde |
| COI vs DLCO | 2-hydroperoxyhexane, 6-heptyn-2-one, 2-methylnonane, 2-butyl-1-octanol | 2-hydroperoxyhexane, 6-heptyn-2-one, benzaldehyde |
| COI vs FEV1 | 2-hydroperoxyhexane, 2-methylnonane, acetophenone | 2-hydroperoxyhexane, acetic acid |
| COI vs FVC | 2-hydroperoxyhexane | - |
| sPLSDA | 6-heptyn-2-one, decane, benzaldehyde, 4-pentenylacetate, acetophenone, 1-fluoroheptane, serine, isoleucine | identified benzaldehyde, 6-heptyn-2-one, 2-hydroperoxyhexane, decane |
| Correlation | All patients | Patients with pulmonary involvement |
| COI vs HRCT score | 2-hydroperoxyhexane, serine, threonine | aspartate |
| COI vs chitotriosidase | 4-pentenyl acetate, 1-fluoroheptane, 2-butyl-1-octanol | 1-fluoroheptane, 4-pentenylacetate |
| COI vs LSM | 4-pentenyl acetate | - |
| COI vs CCL18 | - | 2-hydroperoxyhexane |

**Supplemental table 2 Summary of comparisons and correlations from this study for all participants and the subgroup of patients with pulmonary involvement and their matched controls.** Depicted are the compounds of interest that differed significantly between patients and controls (first line COI analysis) and the compounds of interest for which a significant correlation was found (all other lines). COI: compounds of interest, DLCO: diffusion capacity of the lungs for carbon monoxide, FEV1: forced expiratory volume in 1 second, FVC: forced vital capacity, sPLSDA: sparse partial least squares-discriminant analysis, LSM: lysosphingomyelin, CCL18: chemokine C-C motif ligand 18.
